# Circadian Neuropeptide-Expressing Clock Neurons as Regulators of Long-Term Memory: Molecular and Cellular Perspectives

**DOI:** 10.3389/fnmol.2022.934222

**Published:** 2022-07-13

**Authors:** Show Inami, Tomohito Sato, Takaomi Sakai

**Affiliations:** ^1^Department of Neuroscience, Farber Institute for Neurosciences, Thomas Jefferson University, Philadelphia, PA, United States; ^2^Department of Biological Sciences, Tokyo Metropolitan University, Tokyo, Japan

**Keywords:** pigment-dispersing factor, clock neurons, long-term memory, courtship conditioning, memory consolidation, memory maintenance, *Drosophila*

## Abstract

The neuropeptide pigment-dispersing factor (Pdf) is critically involved in the regulation of circadian rhythms in various insects. The function of Pdf in circadian rhythms has been best studied in the fruitfly, i.e., *Drosophila melanogaster*. *Drosophila* Pdf is produced in a small subset of circadian clock neurons in the adult brain and functions as a circadian output signal. Recently, however, Pdf has been shown to play important roles not only in regulating circadian rhythms but also in innate and learned behaviors in *Drosophila*. In this mini-review, we will focus on the current findings that Pdf signaling and Pdf-producing neurons are essential for consolidating and maintaining long-term memory induced by the courtship conditioning in *Drosophila* and discuss the mechanisms of courtship memory processing through Pdf-producing neurons.

## Introduction

Animals acquire temporary memories through their experience. Under certain conditions, an acquired memory is consolidated into a stable long-term memory (LTM). Once LTM is established in the brain, it is maintained until recall. The fruitfly *Drosophila melanogaster* uses various genetic techniques that has been used to clarify the molecular mechanisms of learning and memory. Many memory genes are expressed in the mushroom body (MB), which is considered to be the *Drosophila* memory center (Davis, 2005; Griffith and Ejima, 2009). Interestingly, the *Drosophila* circadian clock gene *period* (*per*) also plays a vital role in memory consolidation to establish LTM, although *per* is not expressed in MB neurons (Sakai et al., 2004; Donlea et al., 2009; Chen et al., 2012; Suzuki et al., 2022). Thus, *per*-expressing clock neurons should also be essential for *Drosophila* LTM (Suzuki et al., 2022). However, little is known about how clock neurons modulate LTM formed and maintained in MB.

In the *Drosophila* brain, there are about 150 clock neurons (Peschel and Helfrich-Forster, 2011). They are anatomically divided into seven groups as follows: dorsal neurons 1 (DN1), DN2, and DN3, large ventral lateral neurons (l-LNvs), small ventral lateral neurons (s-LNvs), 5th small ventral lateral neurons (5th s-LNvs), and dorsal lateral neurons (LNds) (Peschel and Helfrich-Forster, 2011). *Drosophila Pigment-dispersing factor* (*Pdf* ) encoding a neuropeptide, which is well conserved in insect species, is specifically expressed in s-LNvs and l-LNvs (Renn et al., 1999; Helfrich-Forster, 2005; Peschel and Helfrich-Forster, 2011). Pdf functions in the brain have been well studied in *Drosophila*. Pdf was initially identified as a neuropeptide required to generate circadian behavioral rhythms (Renn et al., 1999). Subsequent studies revealed that Pdf plays a vital role in the circadian network as an intercellular messenger from Pdf-expressing clock neurons (hereafter referred to as Pdf neurons) to other clock neurons (Shafer and Yao, 2014; Yoshii et al., 2016). Thus, Pdf is widely known as a circadian neuromodulator.

Pdf is essential not only for circadian rhythms but also for other behavioral phenomena. A null mutation of *Pdf* (*Pdf*^*01*^) induces a defective geotaxis, which is restored by Pdf expression in Pdf neurons (Mertens et al., 2005). *Pdf receptor* (*Pdfr*) mutant flies also show the *Pdf*^*01*^-like phenotype (Mertens et al., 2005), indicating that Pdf/Pdfr signaling is essential for the *Drosophila* geotaxis. Pdf/Pdfr signaling is also indispensable for behavioral plasticity. When wild-type males are housed together with rivals for 5 d before mating, their mating duration is extended compared with the socially isolated males (Kim et al., 2012). Pdf/Pdfr signaling is also required for the experience-dependent extension of mating duration, and this behavioral plasticity is regulated by centrally expressing *Pdf* and *Pdfr* in a circadian-clock-independent manner (Kim et al., 2012). Furthermore, *Pdf*^*01*^ flies show a decreased ability to establish short-term aversive olfactory memory (aversive STM), although a null mutation of *Pdfr*, which induces arrhythmic locomotor activity, has no effect on aversive STM (Flyer-Adams et al., 2020), suggesting that Pdf signaling has roles different from those in modifying circadian rhythms.

In this article, we summarize our current knowledge about the novel functions of Pdf signaling and Pdf neurons that are identified in *Drosophila* courtship memory (Inami et al., 2020, 2021).

## Genetic Studies in *Drosophila* Courtship Memory

The courtship conditioning paradigm has been used to measure *Drosophila* memory (Siegel and Hall, 1979). In this paradigm, a virgin male and a mated female were placed in a small chamber. In this situation, the males receive stresses such as physical rejection and male-courtship-inhibiting cues from mated females (conditioning). After conditioning, males show courtship suppression even toward virgin females. Conditioning-dependent male courtship suppression is based on memory formation because many memory mutants isolated by olfactory classical conditioning do not show courtship suppression (Griffith and Ejima, 2009). Based on the retention time, courtship memory is classified into at least two phases. When males are conditioned with mated females for 1 h (1 h of conditioning), they establish a short-term memory (STM). Although STM lasts at least for 8 h, it disappears 24 h after 1 h of conditioning (Inami et al., 2021). On the other hand, when single males were conditioned for 7 h (7 h of conditioning), they form LTM, which lasts for at least 5 d (Sakai et al., 2004, 2012). Since 2004, many genes related to LTM in *Drosophila* courtship memory have been identified (Table 1). Similar to *Drosophila* aversive olfactory memory (Margulies et al., 2005; Davis, 2011), it is considered that MB neurons are responsible for courtship LTM because many LTM genes identified in MB neurons were found to play essential roles in consolidating and maintaining courtship LTM (Table 1). On the other hand, the circadian clock does not affect courtship LTM because LTM in mutant flies with a defective circadian clock (e.g., *timeless*^*01*^, *cycle*^*0*^, and *Clock*^*Jrk*^) is intact (Sakai et al., 2004).

**Table 1:** Genes related to courtship LTM in *Drosophila*.

**Gene**	**Function**	**Related brain neurons**	**References**
*CrebB*	Transcription factor	–	Sakai et al., 2004
		MBs (α/β & γ lobes)	Inami et al., 2020
*period*	Circadian clock gene	Clock neurons	Sakai et al., 2004
		Clock neurons	Donlea et al., 2009
		LNds	Suzuki et al., 2022
*Notch*	Transcription factor	MBs	Presente et al., 2004
*Orb2*	*Drosophila* homolog for CPEB	MBs	Keleman et al., 2007
		MBs (γ lobes)	Kruttner et al., 2015
*blistered*	*Drosophila* homolog for serum response factor (SRF)	Clock neurons	Donlea et al., 2009
*Dominant temperature sensitive 3*	Ecdysone synthetic pathway	–	Ishimoto et al., 2009
*Ecdysone receptor*	Ecdysone receptor	MBs	Ishimoto et al., 2009
*Histone deacetylase 1*	Histone deacetylase	MBs	Fitzsimons and Scott, 2011
*small conductance calcium-activated potassium channel*	Potassium channel	PNs	Abou Tayoun et al., 2012
*painless*	TRP channnel	MBs & IPCs	Sakai et al., 2012
*Histone deacetylase 4*	Histone deacetylase	MBs	Fitzsimons et al., 2013
*Pigment-dispersing factor*	Neuropeptide	l-LNvs	Inami et al., 2020
*Pdf receptor*	Neuropeptide receptor	–	Inami et al., 2020
*apterous*	Transcription factor	MBs (α/β lobes) & l-LNvs	Inami et al., 2021
*Chip*	Cofactor of Apterous	MBs (α/β lobes)	Inami et al., 2021
*Resistant to dieldrin*	GABA_A_ receptor	l-LNvs	Inami et al., 2021
*Ecdysis triggering hormone*	Master hormone in ecdysis	–	Lee and Adams, 2021
*ETHR*	Ecdysis triggering hormone receptor	MBs (γ lobes)	Lee and Adams, 2021

*MBs, mushroom bodies; LNds, dorsal lateral clock neurons; l-LNvs, large ventral lateral clock neurons; PNs, olfactory projection neurons; IPCs, insulin-producing cells*.

**Figure 1:**
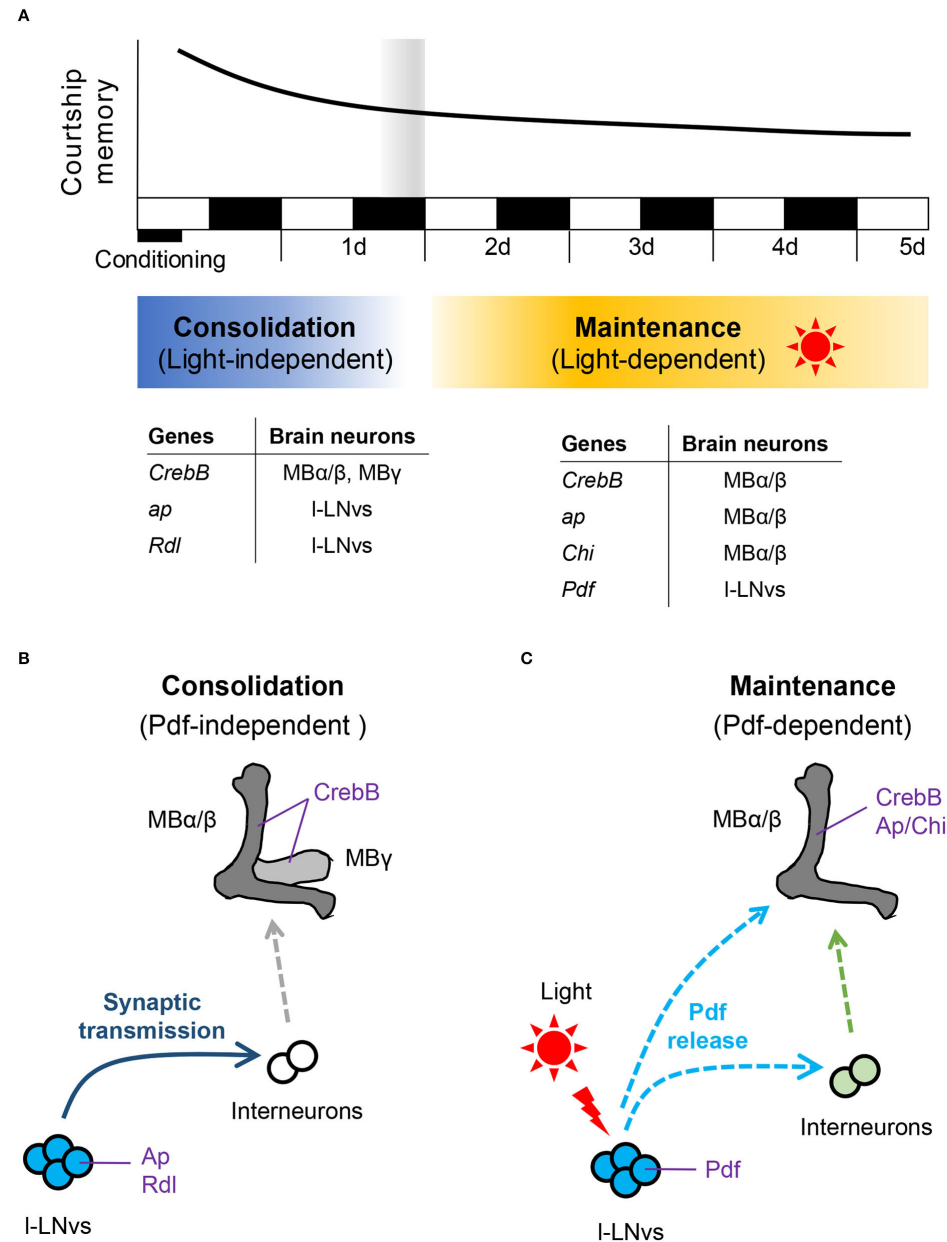
Molecular and cellular basis of courtship memory in *Drosophila*. **(A)** Schematic diagram of courtship memory processing and genes regulating consolidation and maintenance of courtship LTM. *ap, apterous*; *Rdl, Resistant to dieldrin*; *Chi, Chip*; *Pdf*, *Pigment-dispersing factor*. **(B)** Pdf-independent LTM consolidation. Synaptic transmission from Pdf-positive l-LNvs modulates the consolidation of courtship LTM. **(C)** Pdf-dependent LTM maintenance. Pdf release driven by light is essential for the maintenance of courtship LTM.

The cAMP signaling pathways and the transcription factor cAMP response element-binding protein (CREB) are evolutionarily conserved in the vertebrates and invertebrates, and they play critical roles in memory consolidation to establish LTM (Yin and Tully, 1996; Davis, 2005; Kandel, 2012). Thus, synthesis of newly proteins is essential for memory consolidation in vertebrates and invertebrates (Kandel, 2012). In *Drosophila*, synthesis of newly proteins *via* CREB-dependent transcription in MB neurons is indispensable for consolidating and maintaining LTM induced by olfactory classical conditioning and courtship conditioning (Yin and Tully, 1996; Sakai et al., 2004; Ishimoto et al., 2009; Hirano et al., 2016; Inami et al., 2020). In the adult brain, MB neurons comprise of at least three types (α/β, α′/β′, and γ), and each type extends into axonal lobes (α/β, α′/β′, and γ lobes) (Davis, 2005; Mabuchi et al., 2016). Although CREB activity in α′/β′ neurons do not affect the consolidation and maintenance of courtship LTM, CREB activity in α/β and γ neurons during courtship conditioning is necessary for LTM, suggesting that α/β and γ neurons play an essential role in memory consolidation to establish courtship LTM (Inami et al., 2020). However, CREB activity in only α/β neurons, but not that in α′/β′ and γ neurons, is necessary for keeping courtship LTM for more than 2 d (Inami et al., 2020), indicating that the early phase of courtship LTM, which lasts for at least 1 d after conditioning, is formed in α/β and γ neurons, whereas the late phase of courtship LTM, which persists for more than 2 d, is maintained in only α/β neurons (Figure 1A). Thus, courtship memory seems to be consolidated within at least 1 day after conditioning, and the maintenance phase of courtship LTM appears to begin at least 2 d after the courtship conditioning (Figure 1A). However, it still remains unclarified exactly when LTM consolidation ends and how the memory consolidation phase transitions to the LTM maintenance phase.

## Light-Dependent Functions of Pdf Neurons

Various research studies on *Drosophila* chronobiology support the idea that s-LNvs are essential for sustaining circadian locomotor rhythms in constant darkness (Grima et al., 2004; Stoleru et al., 2004, 2005; Helfrich-Forster, 2005; Rieger et al., 2006). Unlike the s-LNvs, l-LNvs mainly contribute to sleep and arousal regulation (Parisky et al., 2008; Shang et al., 2008; Sheeba et al., 2008b; Chung et al., 2009; Shimada et al., 2016). Cryptochrome (Cry), a blue-light-sensitive photopigment, is expressed in many clock neurons containing s-LNvs and l-LNvs, and it acts as a circadian photoreceptor in *Drosophila* (Stanewsky et al., 1998; Emery et al., 2000; Yoshii et al., 2016). Similarly, Rhodopsin 7 (Rh7) also contributes to the light sensitivity of s-LNvs and l-LNvs (Ni et al., 2017). Furthermore, Pdf neurons can sense environmental light directly *via* the circadian photoreceptors Cry and Rh7 (Sheeba et al., 2008a; Fogle et al., 2011; Ni et al., 2017) or indirectly *via* one of the light-sensing organs, the Hofbauer–Buchner (H–B) eyelets (Yoshii et al., 2016; Li et al., 2018). Thus, it is considered that the l-LNvs induce light-dependent Pdf secretion, which regulates light-mediated arousal in *Drosophila* (Shang et al., 2008; Sheeba et al., 2008b).

The rhythmic light–dark (LD) cycles on the Earth significantly affect animal behavior and physiology (Inami et al., 2020). In animals, light is not only essential for acquiring information for image-forming vision in nature but also acts as a potent modulator of brain functions such as circadian entrainment, hormone secretion, sleep–wake cycles, mood, and cognitive functions (Vandewalle et al., 2009; Crocker et al., 2016; Fernandez et al., 2018; Inami et al., 2020). We have recently found that environmental light affects courtship LTM maintenance, but not memory consolidation (Inami et al., 2020). Regardless of whether flies are conditioned in light or darkness, 5-d memory after courtship conditioning is detected. Thus, courtship memory is consolidated into LTM regardless of the presence or absence of light. Unlike memory consolidation, when flies are kept in constant darkness (DD) after the courtship conditioning and before the test, their LTM disappears. Furthermore, DD for 2 d after the conditioning is sufficient to impair LTM. Thus, light is essential for LTM maintenance. Although the amount of daytime sleep in DD is slightly but significantly smaller than that in LD, the decreased sleep amount has no effect on LTM maintenance (Inami et al., 2020). Furthermore, Pdf expression is also critical for the maintenance of courtship LTM (Inami et al., 2020). Temporal activation of Pdf neurons compensates for the DD-inducible LTM impairment. In contrast, l-LNv-specific electrical silencing using the inwardly rectifying Kir2.1 channel impairs LTM maintenance in LD (Inami et al., 2020). Considering these findings, it is most likely that light-inducible Pdf secretion from l-LNvs regulates the light-dependent maintenance of courtship LTM.

A null mutation of *Pdf* does not affect 1-d memory, whereas it impairs 2-d memory or 5-d memory (Inami et al., 2020). These findings support the idea that Pdf release is required for only the maintenance of LTM. If LTM maintenance is light-dependent in *Drosophila*, is CREB activity in MB neurons also light-dependent during the memory maintenance phase? A bioluminescent reporter assay revealed that CREB-dependent transcription in α/β neurons is also light-dependent, but that in α′/β′ and γ neurons is not (Inami et al., 2020). These findings also support the idea that courtship LTM is maintained in α/β neurons in a light-dependent manner.

A null mutation of *Pdfr* also impairs 5-d memory and markedly attenuates a light-dependent increase in the CREB activity in α/β neurons (Inami et al., 2020). Similarly, flies that are kept in DD for 2 d also do not show a light-dependent increase in CREB activity in α/β neurons (Inami et al., 2020). Thus, it is considered that environmental light triggers CREB-dependent transcription in α/β neurons *via* Pdf/Pdfr signaling, and this system is essential for the maintenance of courtship LTM.

The circadian clock drives the rhythmic expression of hundreds of genes in MB neurons, including *Pka-C1*, which encodes a regulatory subunit of cAMP-dependent protein kinase A (PKA) (Almeida et al., 2021). Since CREB phosphorylated by PKA is transcriptionally active (Kandel, 2012), the circadian clock may also regulate CREB activity in MB neurons in DD. However, since CREB activity in MB neurons in LD is markedly higher than that in DD (Inami et al., 2020), the effect of light on CREB activity may outweigh that of the circadian clock.

## Excitability of Pdf Neurons is Essential for Courtship Memory Consolidation

The LIM homeodomain protein Apterous (Ap), which acts as a transcription factor, is well conserved in vertebrates and invertebrates (Hobert and Westphal, 2000). Ap and its cofactor Chip (Chi) are essential for the neuro developmental events (Lundgren et al., 1995; O'Keefe et al., 1998; van Meyel et al., 2000). However, Ap continues to be expressed in the brain neurons including MB α/β neurons, s-LNvs, and l-LNvs (Shimada et al., 2016; Inami et al., 2021). We have recently found that Ap and Chi in MB α/β neurons are indispensable for maintaining courtship LTM (Figure 1A) (Inami et al., 2021). Since Ap/Chi regulates the transcription of Ap target genes (Hobert and Westphal, 2000; Inami et al., 2021), Ap/Chi in MB α/β neurons should be necessary for providing proteins required to maintain courtship LTM (Inami et al., 2021). As was observed in Ap/Chi, CREB-dependent transcription in MB α/β neurons is also essential for the maintenance of courtship LTM. Thus, courtship LTM is likely maintained in MB α/β neurons from the second day after conditioning, and proteins required for maintaining LTM for more than 2 d should be provided *via* transcriptions by CREB and Ap/Chi. However, the molecular interactions between CREB and Ap/Chi still remain unclarified.

Unlike Ap in MB α/β neurons, Ap in l-LNvs, but not in s-LNvs, is essential for memory consolidation to establish courtship LTM in a Chi-independent manner (Inami et al., 2021) (Figure 1B). In addition, Ap in l-LNvs plays a vital role in preventing over-responses to the inhibitory neurotransmitter GABA. The induction of the *Drosophila* ionotropic GABA_A_ receptor on the Pdf neurons compensates for the impaired memory consolidation in *ap* null mutant flies (Inami et al., 2021). These findings indicate that the excitability of Pdf neurons plays a crucial role in memory consolidation to establish LTM.

## Synaptic Transmission From Pdf Neurons is Necessary for Consolidation of Courtship LTM

*Drosophila shibire* (*shi*) encodes Dynamin regulating synaptic vesicle recycling (Vanderbliek and Meyerowitz, 1991). Induction of the temperature-sensitive *shi* allele (*shi*^*ts*1^) can inhibit synaptic transmission in a temperature-dependent manner (Kitamoto, 2001; Suzuki et al., 2022). Although Shi^ts1^ functions as normal Dynamin at the permissive temperature, it is dysfunctional at the restrictive temperature. Thus, the targeted expression of *shi*^*ts*1^ can spatially and temporally inhibit synaptic transmission through a temperature shift (Kasuya et al., 2009). Disruption of synaptic transmission in PDF neurons using *shi*^*ts*1^ impairs memory consolidation. However, it does not affect LTM maintenance or recall. These findings indicate that synaptic transmission in Pdf neurons mainly contributes to memory consolidation (Figure 1B) (Inami et al., 2021). Why does disruption of synaptic transmission in Pdf neurons impair memory consolidation, although the Pdf neuropeptide does not affect memory consolidation? We previously reported that disruption of synaptic transmission in Pdf neurons using *shi*^*ts*1^ has little impact on locomotor activity rhythms (Mabuchi et al., 2016). This finding suggests that disruption of the Dynamin function cannot inhibit Pdf release. Thus, it is likely that neurotransmitters other than Pdf released from Pdf neurons are involved in the consolidation of courtship LTM.

## Discussion

The current research studies using *Drosophila* courtship conditioning reveal that Pdf neurons have two distinct functions and modify two different memory processes. First, dynamin-dependent neurotransmission from Pdf neurons during courtship conditioning is essential for memory consolidation to establish courtship LTM (Figure 1B). Since Pdf neuropeptide release seems to be dynamin-independent, other neurotransmitters such as the classical neurotransmitters should be released from Pdf neurons. However, it remains unknown whether neurotransmission from Pdf neurons is driven in a conditioning-dependent manner or endogenously occurs in Pdf neurons. Since, to the best of our knowledge, there is no direct evidence that l-LNvs synaptically project to MB α/β or γ neurons directly, intercellular communication from l-LNvs to MB α/β and/or γ neurons *via* interneurons may play a crucial role in the establishment of courtship LTM (Figure 1B). Second, the light-dependent release of the Pdf neuropeptide from l-LNvs plays a critical role in the courtship LTM maintenance (Figure 1C). Environmental light induces Pdf release and activates the transcription factor CREB in MB α/β neurons. Moreover, the light dependent CREB activation in MB α/β neurons occurs *via* Pdfr. Chronobiological research studies using *Pdfr*-GAL4 lines or an anti-Pdfr antibody did not indicate Pdfr expression in MB neurons (Mertens et al., 2005; Im and Taghert, 2010). In contrast, RNA sequencing analysis has revealed that *Pdfr* is expressed in MB neurons (Crocker et al., 2016). Furthermore, Flyer-Adams et al. have recently shown using a LexA knock-in fly strain, *Pdfr-2A-LexA* that *Pdfr* is expressed in at least one of the MB neurons (Flyer-Adams et al., 2020). Although it remains to be clarified whether activated Pdfr directly or indirectly increases CREB activity in MB α/β neurons, the light-dependent Pdf/Pdfr/CREB pathway is found to be essential for courtship LTM maintenance (Inami et al., 2020).

In *Drosophila*, the LTM maintenance phase has been defined conceptually as the time after LTM is fully formed and consolidated, and it is generally believed that memory consolidation is completed within 1 d after conditioning (Davis, 2005; Margulies et al., 2005; Inami et al., 2021). The recent LTM research studies using *Drosophila* courtship conditioning identified interesting mutants or transgenic flies with intact 1-d memory but are defective 2-d memory (Inami et al., 2020, 2021). This finding indicates that there are genetically manipulated flies that can consolidate LTM but cannot maintain it. Furthermore, the recent studies showed the vital roles of Pdf neurons in modulating LTM processes in a Pdf-dependent or Pdf-independent manner (Inami et al., 2020, 2021). Considering these findings, the consolidation and maintenance phases in the courtship LTM seem to be molecularly and cellularly separate (Figure 1A). Although it will be necessary to determine whether this model can be extended to other memory paradigms in *Drosophila*, the clock neuron network and the memory center may, in general, cooperatively work in establishing and maintaining *Drosophila* LTM.

## Author Contributions

SI, TSt, and TSk contributed to conception and design of the study. TSk wrote the first draft of the manuscript. SI and TSt wrote sections of the manuscript. All authors contributed to manuscript revision, read, and approved the submitted version.

## Funding

This work was supported by a JSPS KAKENHI (grant number 15J06303) to SI, JSPS KAKENHI (grant numbers 16H04816 and 21H02528) to TSk, and a Grant-in-Aid for Scientific Research on Innovative Areas, Singularity Biology (grant number 21H00434) to TSk.

## Conflict of Interest

The authors declare that the research was conducted in the absence of any commercial or financial relationships that could be construed as a potential conflict of interest.

## Publisher's Note

All claims expressed in this article are solely those of the authors and do not necessarily represent those of their affiliated organizations, or those of the publisher, the editors and the reviewers. Any product that may be evaluated in this article, or claim that may be made by its manufacturer, is not guaranteed or endorsed by the publisher.

## References

[B1] Abou Tayoun A. N. Pikielny C. Dolph P. J. (2012). Roles of the *Drosophila* SK channel (dSK) in courtship memory. PLoS ONE 7, e34665. 10.1371/journal.pone.0034665 22509342 PMC3324495

[B2] Almeida P. M. Solis B. L. Feidler A. Nagoshi E. Stickley L. (2021). Neurofibromin 1 in mushroom body neurons mediates circadian wake drive through activating cAMP-PKA signaling. Nat. Commun. 12, 5758. 10.1038/s41467-021-26031-2 34599173 PMC8486785

[B3] Chen C. C. Wu J. K. Lin H. W. Pai T. P. Fu T. F. Wu C. L.. (2012). Visualizing long-term memory formation in two neurons of the *Drosophila* brain. Science 335, 678–685. 10.1126/science.1212735 22323813

[B4] Chung B. Y. Kilman V. L. Keath J. R. Pitman J. L. Allada R. (2009). The GABA(A) receptor RDL acts in peptidergic PDF neurons to promote sleep in *Drosophila*. Curr. Biol. 19, 386–390. 10.1016/j.cub.2009.01.040 19230663 PMC3209479

[B5] Crocker A. Guan X. J. Murphy C. T. Murthy M. (2016). Cell-type-specific transcriptome analysis in the *Drosophila* mushroom body reveals memory-related changes in gene expression. Cell Rep. 15, 1580–1596. 10.1016/j.celrep.2016.04.046 27160913 PMC5047377

[B6] Davis R. L. (2005). Olfactory memory formation in *Drosophila*: from molecular to systems neuroscience. Annu. Rev. Neurosci. 28, 275–302. 10.1146/annurev.neuro.28.061604.135651 16022597

[B7] Davis R. L. (2011). Traces of *Drosophila* memory. Neuron 70, 8–19. 10.1016/j.neuron.2011.03.012 21482352 PMC3374581

[B8] Donlea J. M. Ramanan N. Shaw P. J. (2009). Use-dependent plasticity in clock neurons regulates sleep need in *Drosophila*. Science 324, 105–108. 10.1126/science.1166657 19342592 PMC2850598

[B9] Emery P. Stanewsky R. Helfrich-Forster C. Emery-Le M. Hall J. C. Rosbash M. (2000). *Drosophila* CRY is a deep brain circadian photoreceptor. Neuron 26, 493–504. 10.1016/S0896-6273(00)81181-2 10839367

[B10] Fernandez D. C. Fogerson P. M. Ospri L. L. Thomsen M. B. Layne R. M. Severin D.. (2018). Light affects mood and learning through distinct retina-brain pathways. Cell 175, 71–84. 10.1016/j.cell.2018.08.004 30173913 PMC6190605

[B11] Fitzsimons H. L. Schwartz S. Given F. M. Scott M. J. (2013). The histone deacetylase HDAC4 regulates long-term memory in *Drosophila*. PLoS ONE 8, e83903. 10.1371/journal.pone.0083903 24349558 PMC3857321

[B12] Fitzsimons H. L. Scott M. J. (2011). Genetic modulation of Rpd3 expression impairs long-term courtship memory in *Drosophila*. PLoS ONE 6, e29171. 10.1371/journal.pone.0029171 22195015 PMC3240647

[B13] Flyer-Adams J. G. Rivera-Rodriguez E. J. Yu J. Mardovin J. D. Reed M. L. Griffith L. C. (2020). Regulation of olfactory associative memory by the circadian clock output signal Pigment-Dispersing Factor (PDF). J. Neurosci. 40, 9066–9077. 10.1523/JNEUROSCI.0782-20.2020 33106351 PMC7673005

[B14] Fogle K. J. Parson K. G. Dahm N. A. Holmes T. C. (2011). CRYPTOCHROME is a blue-light sensor that regulates neuronal firing rate. Science 331, 1409–1413. 10.1126/science.1199702 21385718 PMC4418525

[B15] Griffith L. C. Ejima A. (2009). Courtship learning in *Drosophila melanogaster*: diverse plasticity of a reproductive behavior. Learn. Mem. 16, 743–750. 10.1101/lm.956309 19926779 PMC4419844

[B16] Grima B. Chelot E. Xia R. H. Rouyer F. (2004). Morning and evening peaks of activity rely on different clock neurons of the *Drosophila* brain. Nature 431, 869–873. 10.1038/nature02935 15483616

[B17] Helfrich-Forster C. (2005). Neurobiology of the fruit fly's circadian clock. Genes Brain Behav. 4, 65–76. 10.1111/j.1601-183X.2004.00092.x 15720403

[B18] Hirano Y. Ihara K. Masuda T. Yamamoto T. Iwata I. Takahashi A.. (2016). Shifting transcriptional machinery is required for long-term memory maintenance and modification in *Drosophila* mushroom bodies. Nat. Commun. 7, 13471. 10.1038/ncomms13471 27841260 PMC5114576

[B19] Hobert O. Westphal H. (2000). Functions of LIM-homeobox genes. Trends Genet. 16, 75–83. 10.1016/S0168-9525(99)01883-1 10652534

[B20] Im S. H. Taghert P. H. (2010). PDF receptor expression reveals direct interactions between circadian oscillators in *Drosophila*. J. Comp. Neurol. 518, 1925–1945. 10.1002/cne.22311 20394051 PMC2881544

[B21] Inami S. Sato S. Kondo S. Tanimoto H. Kitamoto T. Sakai T. (2020). Environmental light is required for maintenance of long-term memory in *Drosophila*. J. Neurosci. 40, 1427–1439. 10.1523/JNEUROSCI.1282-19.2019 31932417 PMC7044726

[B22] Inami S. Sato T. Kurata Y. Suzuki Y. Kitamoto T. Sakai T. (2021). Consolidation and maintenance of long-term memory involve dual functions of the developmental regulator Apterous in clock neurons and mushroom bodies in the *Drosophila* brain. PLoS Biol. 19, e3001459. 10.1371/journal.pbio.3001459 34860826 PMC8641882

[B23] Ishimoto H. Sakai T. Kitamoto T. (2009). Ecdysone signaling regulates the formation of long-term courtship memory in adult *Drosophila melanogaster*. Proc. Natl. Acad. Sci. U. S. A. 106, 6381–6386. 10.1073/pnas.0810213106 19342482 PMC2669368

[B24] Kandel E. R. (2012). The molecular biology of memory: cAMP, PKA, CRE, CREB-1, CREB-2, and CPEB. Mol. Brain 5, 14. 10.1186/1756-6606-5-14 22583753 PMC3514210

[B25] Kasuya J. Ishimoto H. Kitamoto T. (2009). Neuronal mechanisms of learning and memory revealed by spatial and temporal suppression of neurotransmission using *shibire*, a temperature-sensitive dynamin mutant gene in *Drosophila melanogaster*. Front. Mol. Neurosci. 2, 11. 10.3389/neuro.02.011.2009 19738923 PMC2737436

[B26] Keleman K. Kruttner S. Alenius M. Dickson B. J. (2007). Function of the *Drosophila* CPEB protein Orb2 in long-term courtship memory. Nat. Neurosci. 10, 1587–1593. 10.1038/nn1996 17965711

[B27] Kim W. J. Jan L. Y. Jan Y. N. (2012). Contribution of visual and circadian neural circuits to memory for prolonged mating induced by rivals. Nat. Neurosci. 15, 876–883. 10.1038/nn.3104 22561453 PMC3417086

[B28] Kitamoto T. (2001). Conditional modification of behavior in *Drosophila* by targeted expression of a temperature-sensitive *shibire* allele in defined neurons. J. Neurobiol. 47, 81–92. 10.1002/neu.1018 11291099

[B29] Kruttner S. Traunmuller L. Dag U. Jandrasits K. Stepien B. Iyer N.. (2015). Synaptic Orb2A bridges memory acquisition and late memory consolidation in *Drosophila*. Cell Rep. 11, 1953–1965. 10.1016/j.celrep.2015.05.037 26095367 PMC4508346

[B30] Lee S. S. Adams M. E. (2021). Regulation of *Drosophila* long-term courtship memory by ecdysis triggering hormone. Front. Neurosci. 15, 670322. 10.3389/fnins.2021.670322 33967686 PMC8100193

[B31] Li M. T. Cao L. H. Xiao N. Tang M. Deng B. Yang T.. (2018). Hub-organized parallel circuits of central circadian pacemaker neurons for visual photoentrainment in *Drosophila*. Nat. Commun. 9, 4247. 10.1038/s41467-018-06506-5 30315165 PMC6185921

[B32] Lundgren S. E. Callahan C. A. Thor S. Thomas J. B. (1995). Control of neuronal pathway selection by the *Drosophila* LIM homeodomain gene apterous. Development 121, 1769–1773. 10.1242/dev.121.6.1769 7600992

[B33] Mabuchi I. Shimada N. Sato S. Ienaga K. Inami S. Sakai T. (2016). Mushroom body signaling is required for locomotor activity rhythms in *Drosophila*. Neurosci Res 111, 25–33. 10.1016/j.neures.2016.04.005 27106579

[B34] Margulies C. Tully T. Dubnau J. (2005). Deconstructing memory in *Drosophila*. Curr. Biol. 15, R700– R 713. 10.1016/j.cub.2005.08.024 16139203 PMC3044934

[B35] Mertens I. Vandingenen A. Johnson E. C. Shafer O. T. Li W. Trigg J. S.. (2005). PDF receptor signaling in *Drosophila* contributes to both circadian and geotactic behaviors. Neuron 48, 213–219. 10.1016/j.neuron.2005.09.009 16242402

[B36] Ni J. D. Baik L. S. Holmes T. C. Montell C. (2017). A rhodopsin in the brain functions in circadian photoentrainment in *Drosophila*. Nature 545, 340–344. 10.1038/nature22325 28489826 PMC5476302

[B37] O'Keefe D. D. Thor S. Thomas J. B. (1998). Function and specificity of LIM domains in *Drosophila* nervous system and wing development. Development 125, 3915–3923. 10.1242/dev.125.19.3915 9729499

[B38] Parisky K. M. Agosto J. Pulver S. R. Shang Y. Kuklin E. Hodge J. J.. (2008). PDF cells are a GABA-responsive wake-promoting component of the *Drosophila* sleep circuit. Neuron 60, 672–682. 10.1016/j.neuron.2008.10.042 19038223 PMC2734413

[B39] Peschel N. Helfrich-Forster C. (2011). Setting the clock–by nature: circadian rhythm in the fruitfly *Drosophila melanogaster*. FEBS Lett. 585, 1435–1442. 10.1016/j.febslet.2011.02.028 21354415

[B40] Presente A. Boyles R. S. Serway C. N. de Belle J. S. Andres A. J. (2004). Notch is required for long-term memory in *Drosophila*. Proc. Natl. Acad. Sci. U. S. A. 101, 1764–1768. 10.1073/pnas.0308259100 14752200 PMC341850

[B41] Renn S. C. P. Park J. H. Rosbash M. Hall J. C. Taghert P. H. (1999). A *pdf* neuropeptide gene mutation and ablation of PDF neurons each cause severe abnormalities of behavioral circadian rhythms in *Drosophila*. Cell 99, 791–802. 10.1016/S0092-8674(00)81676-1 10619432

[B42] Rieger D. Shafer O. T. Tomioka K. Helfrich-Forster C. (2006). Functional analysis of circadian pacemaker neurons in *Drosophila melanogaster*. J. Neurosci. 26, 2531–2543. 10.1523/JNEUROSCI.1234-05.2006 16510731 PMC6793667

[B43] Sakai T. Sato S. Ishimoto H. Kitamoto T. (2012). Significance of the centrally expressed TRP channel painless in *Drosophila* courtship memory. Learn. Mem. 20, 34–40. 10.1101/lm.029041.112 23247253 PMC3533128

[B44] Sakai T. Tamura T. Kitamoto T. Kidokoro Y. (2004). A clock gene, *period*, plays a key role in long-term memory formation in *Drosophila*. Proc. Natl. Acad. Sci. U. S. A. 101, 16058–16063. 10.1073/pnas.0401472101 15522971 PMC528738

[B45] Shafer O. T. Yao Z. (2014). Pigment-dispersing factor signaling and circadian rhythms in insect locomotor activity. Curr. Opin. Insect Sci. 1, 73–80. 10.1016/j.cois.2014.05.002 25386391 PMC4224320

[B46] Shang Y. Griffith L. C. Rosbash M. (2008). Light-arousal and circadian photoreception circuits intersect at the large PDF cells of the *Drosophila* brain. Proc. Natl. Acad. Sci. U. S. A. 105, 19587–19594. 10.1073/pnas.0809577105 19060186 PMC2596742

[B47] Sheeba V. Fogle K. J. Kaneko M. Rashid S. Chou Y. T. Sharma V. K.. (2008b). Large ventral lateral neurons modulate arousal and sleep in *Drosophila*. Curr. Biol. 18, 1537–1545. 10.1016/j.cub.2008.08.033 18771923 PMC2597195

[B48] Sheeba V. Gu H. Sharma V. K. O'Dowd D. K. Holmes T. C. (2008a). Circadian- and light-dependent regulation of resting membrane potential and spontaneous action potential firing of *Drosophila* circadian pacemaker neurons. J. Neurophysiol. 99, 976–988. 10.1152/jn.00930.2007 18077664 PMC2692874

[B49] Shimada N. Inami S. Sato S. Kitamoto T. Sakai T. (2016). Modulation of light-driven arousal by LIM-homeodomain transcription factor Apterous in large PDF-positive lateral neurons of the *Drosophila* brain. Sci. Rep. 6, 37255. 10.1038/srep37255 27853240 PMC5112534

[B50] Siegel R. W. Hall J. C. (1979). Conditioned-responses in courtship behavior of normal and mutant *Drosophila*. Proc. Natl. Acad. Sci. U. S. A. 76, 3430–3434. 10.1073/pnas.76.7.3430 16592682 PMC383839

[B51] Stanewsky R. Kaneko M. Emery P. Beretta B. Wager-Smith K. Kay S. A.. (1998). The *cry*^*b*^ mutation identifies cryptochrome as a circadian photoreceptor in *Drosophila*. Cell 95, 681–692. 10.1016/S0092-8674(00)81638-4 9845370

[B52] Stoleru D. Peng Y. Agosto J. Rosbash M. (2004). Coupled oscillators control morning and evening locomotor behaviour of *Drosophila*. Nature 431, 862–868. 10.1038/nature02926 15483615

[B53] Stoleru D. Peng Y. Nawathean P. Rosbash M. (2005). A resetting signal between *Drosophila* pacemakers synchronizes morning and evening activity. Nature 438, 238–242. 10.1038/nature04192 16281038

[B54] Suzuki Y. Kurata Y. Sakai T. (2022). Dorsal-lateral clock neurons modulate consolidation and maintenance of long-term memory in *Drosophila*. Genes Cells 27, 266–279. 10.1111/gtc.12923 35094465

[B55] van Meyel D. J. O'Keefe D. D. Thor S. Jurata L. W. Gill G. N. Thomas J. B. (2000). Chip is an essential cofactor for apterous in the regulation of axon guidance in *Drosophila*. Development 127, 1823–1831. 10.1242/dev.127.9.1823 10751171

[B56] Vanderbliek A. M. Meyerowitz E. M. (1991). Dynamin-like protein encoded by the *Drosophila shibire* gene associated with vesicular traffic. Nature 351, 411–414. 10.1038/351411a0 1674590

[B57] Vandewalle G. Maquet P. Dijk D. J. (2009). Light as a modulator of cognitive brain function. Trends Cogn. Sci. 13, 429–438. 10.1016/j.tics.2009.07.004 19748817

[B58] Yin J. C. Tully T. (1996). CREB and the formation of long-term memory. Curr. Opin. Neurobiol. 6, 264–268. 10.1016/S0959-4388(96)80082-1 8725970

[B59] Yoshii T. Hermann-Luibl C. Helfrich-Forster C. (2016). Circadian light-input pathways in *Drosophila*. Commun. Integr. Biol. 9, e1102805. 10.1080/19420889.2015.1102805 27066180 PMC4802797

